# Effects of Solvent Evaporation Methods and Short-Term Room Temperature Storage on High-Coverage Cellular Metabolome Analysis

**DOI:** 10.3390/metabo13101052

**Published:** 2023-10-05

**Authors:** Xian Luo, Liang Li

**Affiliations:** 1 The Metabolomics Innovation Centre, Edmonton, AB T6G 2G2, Canada; xluo2@ualberta.ca; 2 Department of Chemistry, University of Alberta, Edmonton, AB T6G 2G2, Canada

**Keywords:** metabolomics, chemical isotope labeling, LC-MS, sample storage, sample shipment

## Abstract

Cellular metabolomics provides insights into the metabolic processes occurring within cells and can help researchers understand how these processes are regulated and how they relate to cellular function, health, and disease. In this technical note, we investigated the effects of solvent evaporation equipment and storage condition on high-coverage cellular metabolomics. We previously introduced a robust CIL LC-MS-based cellular metabolomics workflow that encompasses various steps, including cell harvest, metabolic quenching, cell lysis, metabolite extraction, differential chemical isotope labeling, and LC-MS analysis. This workflow has consistently served as the cornerstone of our collaborative research and service projects. As a core facility catering to users with diverse research needs and financial resources, we have encountered scenarios requiring short-term sample storage. For example, the need often arises to transport samples at room temperature from user sites to our core facility. Herein, we present a study in which we compared different solvent evaporation methods (specifically, the nitrogen blowdown evaporator, SpeedVac concentrator, and lyophilizer) and diverse storage conditions (including dried samples stored in a freezer, samples stored in a freezer with methanol, dried samples stored at room temperature, and samples stored at room temperature with methanol). Our findings indicate that the choice of solvent evaporation equipment did not significantly impact the cellular metabolome. However, we observed a noteworthy change in the metabolome after 7 days of storage when cells were stored with methanol, regardless of whether they were kept at −80 °C or room temperature, in contrast to cells that were dried and frozen. Importantly, we detected no significant alterations in cells that were dried and stored at room temperature. In conclusion, to ensure the production of high-quality CIL LC-MS metabolomics results, we strongly recommend that, in situations where low-temperature storage is not feasible, cell samples should be thoroughly dried before storage or shipment at room temperature.

## 1. Introduction

Chemical Isotope Labeling (CIL) LC-MS is a powerful method for comprehensive metabolome analysis, with high metabolic coverage and high relative-quantification accuracy [1,2,3,4,5,6,7,8]. Cellular metabolomics involves the comprehensive detection and quantification of a wide range of metabolites within both cells and their growth medium. Cellular metabolomics is widely employed in developing new drugs, identifying disease biomarkers, optimizing biotechnology processes, enhancing production in fermentation engineering, and investigating toxicology mechanisms [9,10,11,12]. For cellular metabolome analysis, pre-analytical process, instrumental analysis and data processing are the three main steps. With high coverage and accuracy, small variations in pre-analytical process can affect the metabolomic results significantly in CIL LC-MS. The major pre-analytical processes of cellular metabolomics include cellular metabolism quenching, cell harvest, sample storage, and sample transport [1,2,13,14,15,16]. We have reported a robust workflow for handling mammalian cells for CIL LC-MS metabolomic profiling [4]. In this workflow, cellular metabolism are quenched by cold methanol, and harvested by scraping. The quenching solvent is removed using a SpeedVac concentrator, and cells are lysed and extracted with freeze–thaw cycles. This method has been successfully applied for CIL LC-MS-based metabolomic profiling of different types of mammalian cells in collaborative research and service projects.

As a core facility supporting metabolomics research, we have worked with many users of different research and financial capabilities around the world. Through these interactions, we have encountered some new issues. For instance, a SpeedVac concentrator is not always accessible in user labs. A lyophilizer is more widely used for drying samples in biological labs. However, methanol, which is used for metabolism quenching, is not compatible with a lyophilizer due to its extremely low freezing point. In addition, some users may only access a certain type of solvent evaporation equipment, such as a nitrogen blowdown concentrator. Unfortunately, we currently do not know the potential impact of using different solvent evaporation equipment on the cellular metabolome. Therefore, comparing different equipment for drying samples can be very helpful in developing a standard operating procedure for preparing cell samples for CIL LC-MS.

Another important issue is related to the means of sample transport from a user lab to a core facility. Ideally, the cell pellets should be shipped with dry ice to keep them frozen. However, dry-ice shipment service is not always available in some countries or regions. Moreover, the cost of dry-ice shipment may not be acceptable for some labs. Shipping samples with dry ice involves potential hazards. Dry ice, in the process of sublimation, releases carbon dioxide (CO_2_) gas. In confined spaces, the accumulation of CO_2_ can displace oxygen, thereby posing a significant asphyxiation risk. Additionally, CO_2_ gas leads to a buildup of pressure within sealed containers. Inadequate venting or pressure control measures can result in container ruptures or, in extreme cases, explosions. Therefore, studying the metabolome alterations that may occur during room temperature shipment should be useful in controlling this pre-analytical variable.

**Figure 1:**
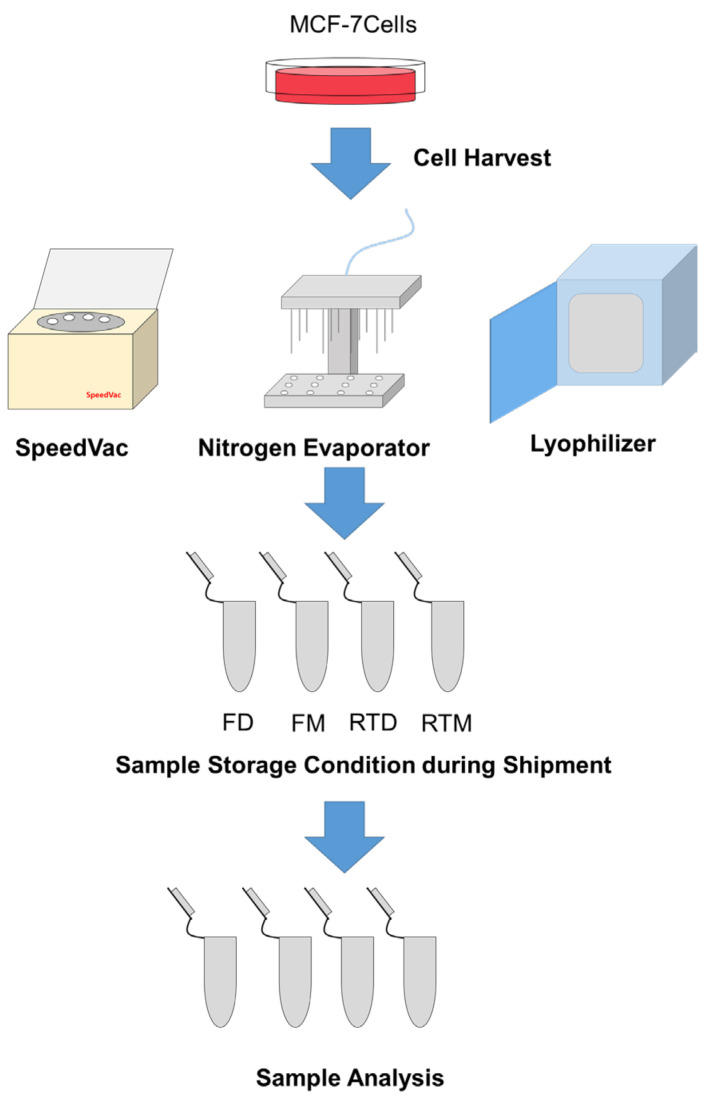
Workflow of evaluating effects of pre-analytical processes on high-coverage cellular metabolomics. FD: dried samples stored in freezer. FM: samples stored in freezer with methanol. RTD: dried samples stored at room temperature. RTM: samples stored at room temperature with methanol.

Figure 1 is the workflow of our study. In this research, we first evaluated the metabolome of cells that were dried by three different types of equipment, i.e., SpeedVac concentrator, nitrogen blowdown concentrator, and lyophilizer. We then compared the metabolome of cells that were stored at four different conditions. These conditions mimicked the dried or non-dried cells, and were shipped with or without dry ice. After performing uni- and multivariate statistical analyses to compare the metabolomic results, we determined the best practice to handle and ship cell samples.

## 2. Materials and Methods

### 2.1. Chemicals and Reagents

All the chemicals and reagents used in this work, unless otherwise stated, were purchased from Fisher Scientific (Hamton, NH, USA). HPLC grade water was purchased from Honeywell (Charlotte, NC, USA). ^12^C- and ^13^C-dansyl chloride were from Nova Medical Testing Inc. (NovaMT) (www.novamt.com) (accessed on 3 February 2023).

### 2.2. Cell Culture

MCF-7 breast cancer cells (HTB-22) were cultured in 6-well plates with 10% FBS supplemented DMEM growth medium. The culture plates were incubated in a 37 °C incubator, with 5% CO_2_ atmosphere. The growth medium was renewed every two days. Biological triplicates were conducted, with approximately 1 million cells utilized for each replicate. Cells were rinsed with cold PBS before harvest. For cells dried using a nitrogen blowdown evaporator (Allsheng, MD-200, Hangzhou, China) or SpeedVac vacuum concentrator (Savant, SC110A, Waltham, MA, USA), 1 mL of cold methanol was added into the wells to quench the cellular metabolism. For cells dried using a lyophilizer (Labconco, Kansas City, MO, USA), 1 mL of cold acetonitrile, instead of methanol, was used to quench the cells. The cells were scraped off the plates and transferred into vials with solvents. Additional 1 mL of solvents were added to rinse the culture plate, and transferred and combined with the quenching solvents, accordingly. The samples were dried using a nitrogen blowdown evaporator (BL), SpeedVac vacuum concentrator (SV), and lyophilizer (LP), respectively.

For storage condition experiments, the harvested cells were stored in a −80 °C freezer or at room temperature for 7 days, with or without methanol. “FD”, “FM”, “RTD”, and “RTM” represented “dried samples stored in freezer”, “samples stored in freezer with methanol”, “dried samples stored at room temperature”, and “samples stored at room temperature with methanol”, respectively. 

### 2.3. Sample Preparation

100 μL of 1:1 MeOH:H_2_O was added into vials containing cell pellets. The vials were frozen in liquid nitrogen, and thawed in a water bath. Five freeze–thaw cycles were performed to ensure that the cells lysed completely. The vials were centrifuged and supernatants were dried down. The dried cell extracts were re-dissolved in 50 μL water. The total metabolite concentration of each sample was determined using a Nova MT Metabolomics Normalization Kit (Edmonton, AB, Canada). Each sample was diluted to 2 mM before chemical isotope labeling. The pooled sample was generated by mixing an equal volume of each individual sample.

### 2.4. Chemical Isotope Labeling

Chemical isotope labeling was carried out by following the user manual of NovaMT CIL Kit (Edmonton, AB, Canada). In brief, 12.5 μL of buffer solution and 37.5 μL of ^12^C-/^13^C-dansyl chloride was added to 25 μL of individual or pooled samples, respectively. The samples were incubated at 40 °C for 45 min to allow the reaction to complete. Then, 7.5 μL of quenching reagent was added to the samples and incubated for 10 min to quench the reaction, and the samples were acidified using a pH adjustment reagent. The ^12^C-labeled individual samples and ^13^C-labeled pool were mixed at an equal volume. The QC sample was generated by combining equal volumes of ^12^C-labeled and ^13^C-labeled pools. The blank sample was generated by mixing of ^12^C-labled water and the ^13^C-labeled pool.

### 2.5. LC-MS Analysis

The samples were injected onto a ZORBAX Eclipse C18 Column (2.1 × 150 mm, 1.8 μm, Agilent, Santa Clara, CA, USA). The QC was injected for every 10 samples to monitor the performance of the LC-MS system. The RT Calibrant (NovaMT, Edmonton, AB, Canada) was injected for every 10 samples. The RT Calibrant consisted of 26 dansyl-labeled standards with different retention times. The LC-MS system was Agilent 1290 UHPLC linked with an Agilent 6546 high resolution Q-TOF mass spectrometer. The mobile phase A was 0.1% formic acid in water, and mobile phase B was 0.1% formic acid in acetonitrile. The gradient was as follows: 0 min, 25% B; 13 min, 99% B; 15 min, 99% B; 15.1 min, 25% B; and 18 min, 25% B. The flow rate was 0.4 mL/min. The autosampler temperature was set at 6 °C, and the column compartment temperature was set at 40 °C. The mass spectrometer settings were as follows: ion polarity, positive; dry gas temperature, 325 °C; capillary voltage, 4000 V; mass range, 100–1000; and acquisition rate, 1 Hz.

### 2.6. Data Processing, Metabolite Identification, and Statistical Analysis

The raw LC-MS data were exported as .csv files. The .csv files were loaded onto IsoMS Pro (NovaMT, Edmonton, AB, Canada). Data quality, including retention time and mass accuracy, was checked. ^12^C/^13^C peak pairs were extracted, and noise peaks were filtered. The multiple LC-MS runs were aligned together by retention time and mass, and missing values were filled. The retention time was corrected based on RT Calibrant before the library search. The metabolite was identified or matched by searching against CIL (Tier 1), Linked ID (Tier 2), and MyCompoundID (MCID) (Tier 3) libraries. The CIL library contained the retention time and accurate mass information of dansyl-labeled standards. The Linked ID library contained predicted retention times and accurate mass information of more than 9000 metabolic pathway-related metabolites. The MCID library contained accurate mass information of approximately 8000 endogenous metabolites and their predicted metabolic products (1 or 2 metabolic reaction). Multivariate (PCA, heatmap) and univariate (volcano plot) analyses were performed using IsoMS Pro 1.2.20.

## 3. Results and Discussion

### 3.1. Cell Line

Comprehensive comparison of cells with different metabolic conditions may offer more valuable insights. However, our primary objective is to offer a more generalized comparison of different solvent evaporation equipment and storage conditions that are applicable to all kinds of cell samples. This approach aims to provide practical insights for researchers and practitioners seeking guidance on sample handling and storage within metabolomics workflows.

Additionally, in our previous study, we observed that different cell lines usually exhibited similar trends in metabolome changes when handled with different pre-analytical methods [4]. Therefore, we exclusively used MCF-7 cells with the same metabolic condition in this study.

### 3.2. Metabolite Identification

In total, 1633 peak pairs were detected from the samples. Of these, 131 peak pairs were identified as Tier 1 metabolites, 231 peak pairs were putatively identified as Tier 2 metabolites, and 1092 peak pairs were matched as Tier 3 metabolites. Overall, 89% of metabolites were identified or matched with different tiers of libraries. The metabolite identification information can be found in Supplemental Table S1. The ID results demonstrated a good metabolome coverage and identification rate of dansylation CIL LC-MS targeting the amine/phenol submetabolome [4].

### 3.3. Solvent Evaporation Equipment Selection

**Figure 2:**
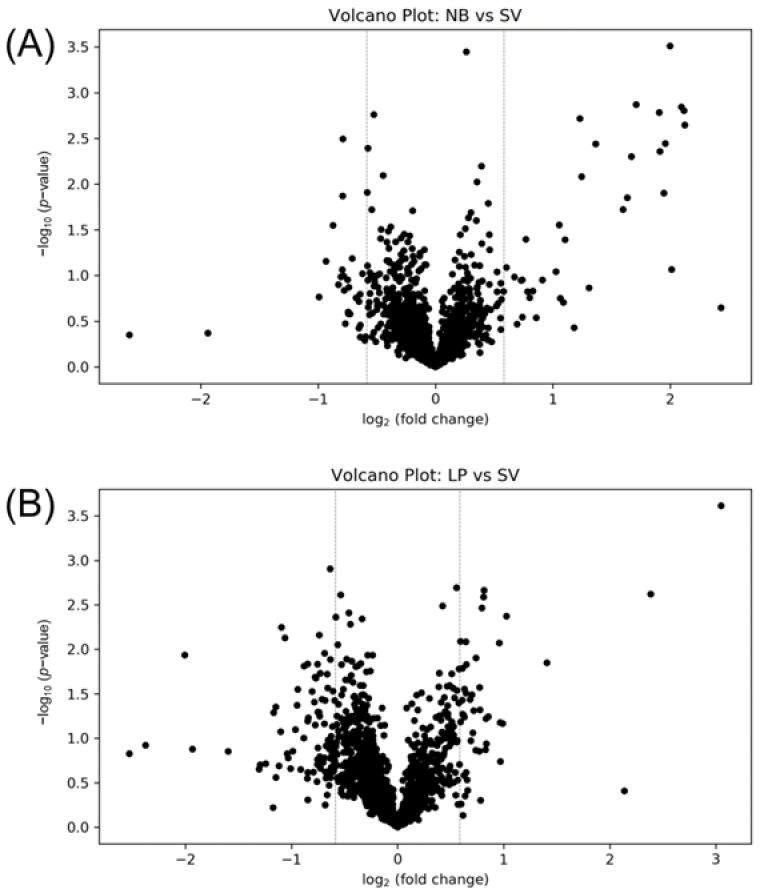
Volcano plot of the metabolome data for the comparison of (**A**) nitrogen blowdown evaporator vs. SpeedVac concentrator and (**B**) lyophilizer vs. SpeedVac concentrator. The dashed lines indicate the threshold of fold change.

Volcano plots were generated to investigate the altered metabolites among the different solvent removing equipment. SpeedVac concentrator (SV) was used to dry the cell pellets in our previous published protocol, and was thus selected as the reference group in generating the volcano plot [4]. Figure 2A shows the comparison of samples dried with the nitrogen blowdown evaporator (NB) and SV. The fold change threshold was 1.5, and the FDR adjustment *p*-value criteria (q-value) was 0.25. In this study, the criterion where the q-value was less than 0.25 was employed for each comparison, instead of other smaller q-values, to maintain a balance of good sensitivity for determining the significantly changed metabolites and good specificity for controlling the false discovery rate. There were no significantly changed metabolites between the NB and SV groups, indicating very small differences between the drying methods using the SpeedVac and nitrogen blowdown evaporators.

Lyophilizer is another widely used equipment for drying down biological samples. Biological samples are frozen and placed in a vacuum chamber, and solvent is slowly removed by sublimating during lyphilizing process. However, using lyophilizer to dry down the organic-solvent-quenched cell line is sometimes challenging. In most of cellular metabolomics studies, methanol is selected as quenching solvent to quench cellular metabolism [13,17]. However, the minimal temperature that a regular lyophilizer can reach is −84 °C, and thus methanol (−98 °C) cannot be frozen in a lyophilizer. Although methanol can be diluted by water and reach a higher freezing point, the collector of lyophilizer is still not cold enough to capture methanol vapor. Uncaptured methanol vapor can contaminate the mechanical pump oil and cause serious environmental and health issues, if not vented properly. The temperature of some uniquely designed lyophilizers can reach as low as −105 °C, but usually cost a lot more. Therefore, to use lyophilizer to dry down the cell line samples, we have to select another organic solvent with a higher freezing point. Acetonitrile has been demonstrated as an efficient quenching reagent, and also has a higher freezing point (−45 °C). It is noteworthy that the structure of enzymes can be rapidly altered by disrupting hydrophobic interaction with many organic solvents, leading to an immediate cessation of metabolic functions [18]. Thus, acetonitrile was used for quenching the cells before lyophilizing in this study. Figure 2B shows the volcano plot of LP vs. SV. The criteria used were the same as for NB vs. SV. No significant change was observed in the volcano plot, indicating that no significant metabolome alternation happened during lyophilizing, compared to SV.

The above results indicate that no significant difference in the cellular metabolome was observed, whether the cell pellets were dried using the nitrogen blowdown evaporator, SpeedVac evaporator, or lyophilizer. Therefore, metabolomics researchers can use any of the equipment to dry down their cell line samples, whichever is accessible. To help researchers in the metabolomics community choose suitable equipment for handling their cell samples, we list the pros and cons of different solvent evaporation equipment in Table 1. The drying speed of the nitrogen evaporator and SpeedVac evaporator was faster than the lyophilizer. The nitrogen evaporator and SpeedVac took ~1 h to remove 2 mL methanol. However, at least 1 day was required for the lyophilizer to dry down the samples with the same volume of solvent. The speed of the nitrogen evaporator could be even higher if higher-flow nitrogen gas is used. Nitrogen blowdown evaporator usually has a lower cost than SpeedVac concentrator and lyophilizer. However, if the nitrogen gas cylinder is used as a gas source, the consumable cost is higher than SpeedVac and lyophilizer. As nitrogen generators are usually installed in mass spectrometry labs, researchers can split gas flow from a nitrogen generator to the blowdown evaporator, which can reduce the consumable cost.

### 3.4. Storage Condition

Cell pellets were stored at different conditions in this study, i.e., cell pellets with methanol were stored at room temperature (RTM) or in a −80 °C freezer (FM), and dried cell pellets were stored at room temperature (RTD) or −80 °C (FD). These four storage conditions mimic four different scenarios. FD is the ideal scenario: the cells are dried and shipped with dry ice. FM represents a lab that cannot access a solvent evaporation equipment, but ship the samples with dry ice. RTD represents a scenario where the cells are dried, but dry ice shipment is not available, or the cost of dry-ice shipment is not acceptable. RTM is the worst scenario, where neither drying equipment or dry ice shipment are accessible. The storage time in this study was set as one week, because for domestic or international shipment the guaranteed delivery time of most couriers is less than 7 days.

**Figure 3:**
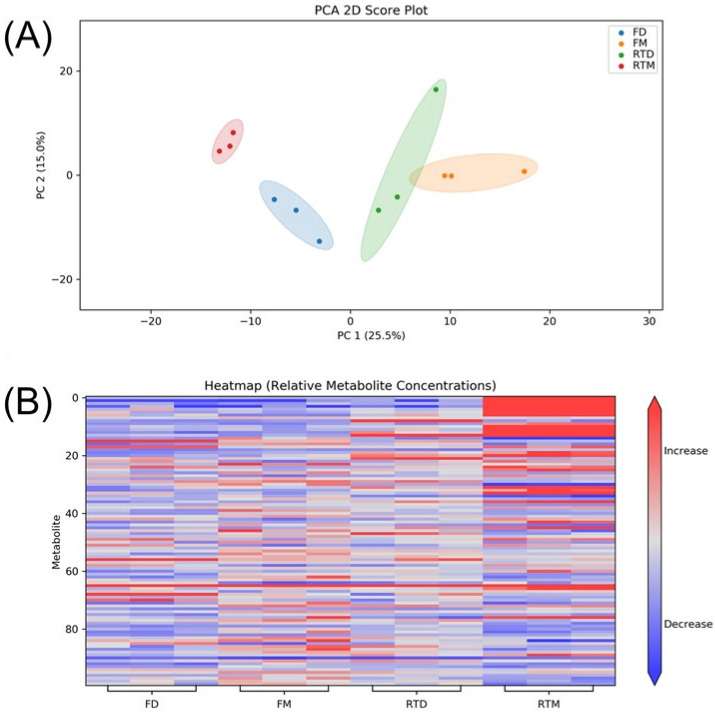
(**A**) PCA plot and (**B**) heatmap of cells stored at different conditions. FD: dried samples stored in freezer. FM: samples stored in freezer with methanol. RTD: dried samples stored at room temperature. RTM: samples stored at room temperature with methanol.

**Figure 4:**
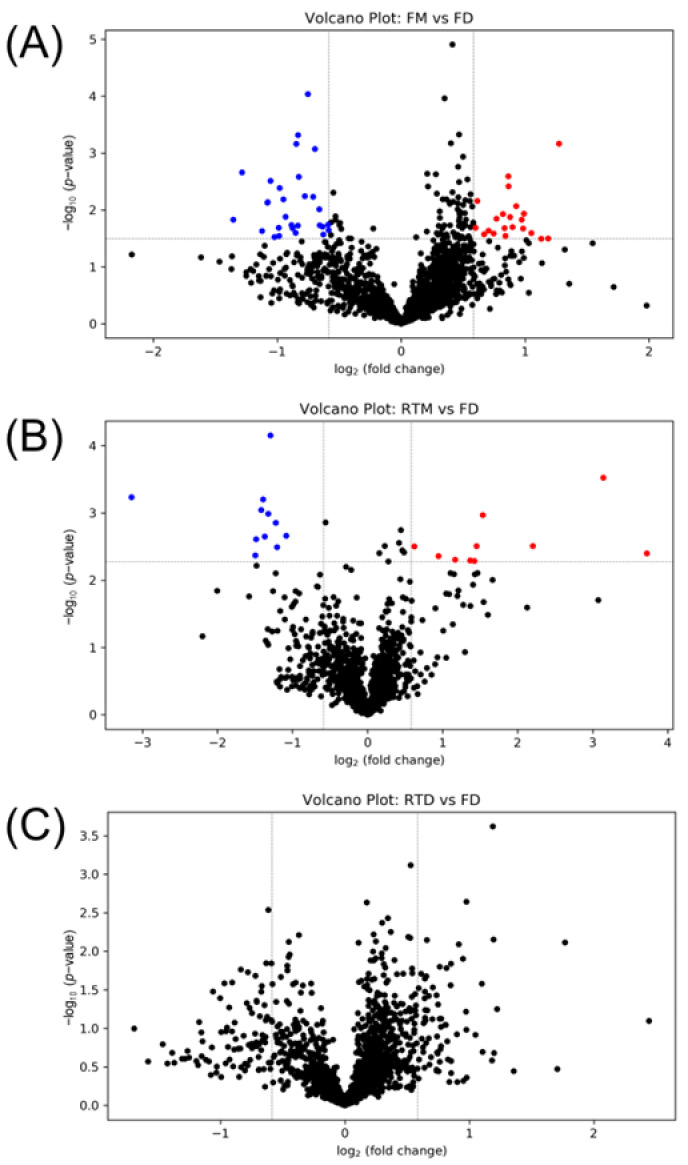
Volcano plot of (**A**) FM vs. FD, (**B**) RTM vs. FD and (**C**) RTD vs. FD. FD: dried samples stored in freezer. FM: samples stored in freezer with methanol. RTD: dried samples stored at room temperature. RTM: samples stored at room temperature with methanol. The dashed lines indicate the threshold of fold change. The red dots represent significantly increased metabolites and blue dots represent significantly decreased metabolites.

Figure 3A shows the PCA plot of cells stored at different conditions. Four groups (FD, FM, RTD, RTM) show clear separation on the PCA plot. The heatmap shown in Figure 3B can be used to visualize metabolite levels in the four different groups. It is clear that some metabolites were significantly increased in the RTM group (top right of the heatmap). Univariate analysis was carried out to further discover the altered metabolites. As dried cell pellets stored in a freezer is considered to be an optimal storage condition, the FD group was selected as a reference group in the volcano plot analysis. Figure 4 shows the volcano plots of FM vs. FD, RTM vs. FD, and RTD vs. FD binary comparisons.

Comparing FM to FD, 21 and 29 metabolites were significantly increased and decreased (Supplemental Table S2). The identified metabolites with an increased level included hydroxyphenyllactici acid (Tier1), isoeugenitol (Tier 2), indoxyl (Tier 2), and N2-acetyl-L-hydroxylysine (Tier 2). The identified metabolites with decreased levels included guanidinoethyl sulfonate (Tier 1), 2,4-diaminotoluene (Tier 2), 2,6-dihydroxypyridine (Tier 2), and gentisate aldehyde 5-O-glucuronide (Tier 2).

Comparing RTM to FD, 10 and 11 metabolite levels were significantly increased and decreased, respectively (Supplemental Table S3). Among them, the levels of glycyl-cysteine (Tier 2), O-acetyl-L-homoserine (Tier 2), and vinyl-acetylglycine (Tier 3, 0 reaction) were increased. In particular, glycyl-cysteine increased 8.8-fold, and O-acetyl-L-homoserine increased 4.6-fold. Unfortunately, none of the metabolites with decreased levels could be identified as Tier 1, 2 and Tier 3 (0 reaction) metabolites.

The changed metabolites belonged to different classes (dipeptides, amino acids derivatives, phenylpropanoic acids, benzenoids, etc.). Thus, it is difficult to predict the metabolite changing patterns during storage. Some of the metabolite level alterations can be explained. For instance, the increased level of dipeptides and amino acid derivatives may be due to the degradation of proteins. The decreased level of some other metabolites may be due to the degradation of metabolites via chemical reactions during storage. For instance, having a similar chemical structure to aniline, 2,4-diaminotoluene is notably susceptible to oxidization. Fortunately, no significantly changed metabolites were observed in the RTD group (Figure 4C).

The results above indicate that if cell pellets are stored with methanol either at −80 °C or room temperature, metabolite level alterations can happen. The effect of dryness is more important than temperature in the sample storage for cellular metabolomic profiling. Therefore, after harvest, the cells should be dried down immediately using any of the accessible equipment in the lab, if room temperature storage or shipment is needed.

## 4. Conclusions

In this study, we investigated the effects of the use of different solvent evaporation equipment and different storage conditions on cellular metabolome results in CIL LC-MS. We conclude that SpeedVac, nitrogen blowdown concentrator, and lyophilizer are all suitable for drying down cell pellets. The results from the sample storage experiment show that metabolite level alterations can happen during the storage of samples containing methanol, whether at room temperature or in a −80 °C freezer. Therefore, the dryness of cell pellets is more important than temperature. If dry-ice shipment is not possible, drying down the cell line samples before shipment is strongly recommended.

It is important to acknowledge that this study has certain limitations. First, we did not assess how varying levels of humidity might affect the metabolome of dried cells. Humidity levels can vary significantly among different laboratories and during the shipment process, potentially influencing metabolite stability. Additionally, the study focused on investigating the impact of short-term storage (7 days) on cellular metabolome. However, we recognize that extreme circumstances can arise, such as significant delays in shipments due to weather or other unforeseen factors, but longer-term sample stability was not examined in this study. In future research, we plan to conduct experiments to investigate the impact of humidity levels and extended storage durations on cellular metabolomes. This will provide a more comprehensive understanding of the factors that can affect metabolite stability during sample storage and shipment.

## Figures and Tables

**Table 1:** Comparison of different solvent evaporation equipment.

	Speed *	Equipment Cost	Consumable Cost
Nitrogen Blowdown Evaporator	~an hour	~USD 2000	~USD 300 per week (nitrogen gas from high pressure cylinder) or ~USD 0 (nitrogen gas from nitrogen generator)
SpeedVac Concentrator	~an hour	~USD 20,000	N/A
Lyophilizer	~one day	~ USD 50,000	N/A

* Speed is defined as the time it takes for 2 mL of methanol or acetonitrile to dry down.

## Data Availability

Data is not publicly available due to privacy. All datasets are available upon request from the authors.

## Supplementary Materials

The following supporting information can be downloaded at: https://www.mdpi.com/article/10.3390/metabo13101052/s1, Supplemental Table S1: List of metabolites identified with different confidence levels; Supplemental Table S2: List of significantly changed metabolites in the comparison of FM vs. FD; Supplemental Table S3: List of significantly changed metabolites in the comparison of RTM vs. FD.
